# Screening of melatonin, α-tocopherol, folic acid, acetyl-l-carnitine and resveratrol for anti-dengue 2 virus activity

**DOI:** 10.1186/s13104-018-3417-3

**Published:** 2018-05-16

**Authors:** Atchara Paemanee, Atitaya Hitakarun, Sittiruk Roytrakul, Duncan R. Smith

**Affiliations:** 1 Molecular Pathology Laboratory, Institute of Molecular Biosciences, Mahidol University, Salaya Campus, 25/25 Phuttamonthol Sai 4, Salaya, Nakorn Pathom 73170 Thailand; 2 Proteomics Research Laboratory, Genome Technology Research Unit, National Center for Genetic Engineering and Biotechnology, National Science and Technology Development Agency, 113 Thailand Science Park, Phahonyothin Road, Khlong Nueng, Khlong Luang, Pathumthani, 12120 Thailand

**Keywords:** Flavivirus, Dengue virus, Melatonin, α-Tocopherol, Folic acid, Acetyl-l-carnitine, Resveratrol

## Abstract

**Objective:**

Infections with the mosquito transmitted dengue virus (DENV) are a significant public health burden in many parts of the world. Despite the introduction of a commercial vaccine in some parts of the world, the majority of the populations at risk of infection remain unprotected against this disease, and there is currently no treatment for DENV infection. Natural compounds offer the prospect of cheap and sustainable therapeutics to reduce the disease burden during infection, and thus potentially alleviate the risk of more severe disease. This study evaluated the potential anti-DENV 2 activity of five natural compounds namely melatonin, α-tocopherol, folic acid, acetyl-l-carnitine and resveratrol in two different cell lines.

**Results:**

Screening of the compounds showed that one compound (acetyl-l-carnitine) showed no effect on DENV infection, three compounds (melatonin, α-tocopherol and folic acid) slightly increased levels of infection, while the 5th compound, resveratrol, showed some limited anti-DENV activity, with resveratrol reducing virus output with an EC_50_ of less than 25 μM. These results suggest that some commonly taken natural compounds may have beneficial effects on DENV infection, but that others may potentially add to the disease burden.

**Electronic supplementary material:**

The online version of this article (10.1186/s13104-018-3417-3) contains supplementary material, which is available to authorized users.

## Introduction

Dengue is currently considered as the most important arthropod-borne viral disease [1]. It is caused by dengue virus (DENV) which is transmitted by female *Aedes* mosquitoes, particularly *Aedes aegypti* [2]. Using the population data of 2010 and a geostatistical model, Bhatt and colleagues estimated that approximately 390 million dengue infections occur globally every year, with 96 million symptomatic cases [3]. Other studies have estimated that more than 22,000 deaths are reported annually [4]. Overall, an estimated 3.9 billion people are at risk of dengue virus infection in almost 128 countries in tropical and subtropical regions [5]. Dengue infection can occur with one of four DENV serotypes (DENV 1–4), and cause clinical manifestations ranging from self-limiting febrile illness, dengue fever to life-threatening dengue hemorrhagic fever which can progress to dengue shock syndrome (DSS) [2]. There is no specific drug to treat DENV infection, and care is mainly supportive [6]. Studies have shown a relationship between viral burden and disease severity [7], and thus there is interest in compounds that have anti-DENV effects, as these may serve to reduce severity if administered early in infection.

De novo drug discovery is both prohibitively expensive [8] and time consuming, and so studies in a wide range of fields have started to explore natural compounds as a way of cutting short the drug development process and additionally developing therapies that are more affordable. This study investigated the anti-DENV effects of 5 commonly used natural compounds that have been shown to have activity against other viruses, or to have the potential to have anti-viral activity, namely melatonin, α-tocopherol, folic acid, acetyl-l-carnitine and resveratrol.

## Main text

### Materials and methods

#### Compounds

Acetyl-l-carnitine (MW 239.70, ≥ 99% purity; Cat.No. A6706, Merck KGaA, Darmstadt, Germany) was prepared as a stock solution of 50 mM in Dulbecco’s modified Eagle’s medium (DMEM, Merck KGaA, Darmstadt, Germany), 10% FBS. Melatonin (MW 232.2, ≥ 98% purity; Cat.No. 444300, Merck KGaA) was prepared as a stock solution of 200 mM in 1 ml of 100% DMSO. α-Tocopherol (MW 430.71, ≥ 95.5% purity, Cat.No. 258024, Merck KGaA) was prepared as a stock solution of 100 mM in EtOH. Folic acid (MW 441.4, ≥ 97% purity, Cat.No. F7876, Merck KGaA) was prepared as a stock solution of 50 mM in 1 M NaOH. Resveratrol (MW 228.2, ≥ 98% purity, Cat No 554325, Merck KGaA) was prepared as a stock solution of 100 mM in 100% DMSO.

#### Cell culture and virus

The human hepatocellular carcinoma cell line HepG2 (ATCC^®^ HB-8065™) and the human embryonic kidney cell line HEK293T/17 (ATCC^®^ CRL-11268™) were cultivated in Dulbecco’s modified Eagle’s medium (DMEM, Merck KGaA) supplemented with 10% heat inactivated fetal bovine serum (FBS, FBS; Gibco, Merck KGaA), 100 U/ml penicillin and 100 µg/ml streptomycin (PAA Laboratories, Pasching, Austria) in a 175 cm^2^ tissue culture flask at 37  °C in 5% CO_2_. Rhesus monkey kidney cells LLC-MK2 (ATCC^®^ CCL-7™) cells were cultivated in DMEM supplemented with 5% FBS and 100 units/ml of penicillin and 100 μg/ml of streptomycin at 37 °C in an incubator with 5% CO_2_. C6/36 (Aedes albopictus, ATCC^®^ CRL-1660™) cells were cultivated in minimum essential medium (MEM; Merck KGaA) supplemented with 10% FBS, 100 units/ml of penicillin and 100 μg/ml of streptomycin at 28 °C. DENV 2 (strain 16681) was propagated in C6/36 cells and the viral titer was determined by standard plaque assay in LLC-MK2 cells essentially as described elsewhere [9].

#### Determination of cytotoxicity

Cytotoxicity of the natural compounds in the range 0.0001–10 mM (Melatonin, ALCAR and α-tocopherol) or 0.0001–5 mM (folic acid and resveratrol) was evaluated by the MTT assay (Merck KGaA) both in infected and uninfected cells.

#### Virus infection for compound screening

HepG2 and HEK293T/17 cells were seeded in 12-well culture plates at a density that allowed 90% confluence to be reached within 24 h. The cells were washed in 1× PBS and incubated with 500 µl of DENV 2 containing the desired multiplicity of infection (MOI) for 2 h. HepG2 cells were infected at MOI 2 and 5, while HEK293T/7 cells were infected at MOI 0.5 and 2 at 37 °C. Mock infection (no virus) was undertaken in parallel. After 2 h of virus infection, medium was replaced with 2 concentrations of each compound in DMEM with 10% FBS and then incubated at 37 °C, 5% CO_2_. After 24-h incubation, cells were collected for determination of the degree of infectivity. For some compounds, cell supernatant was collected and levels of virus production determined by standard plaque assay.

#### Flow cytometry

Flow cytometry to determine percentage cell infection was undertaken using a pan specific anti-dengue E protein antibody HB114 [10] as a primary antibody and a goat anti mouse IgG conjugated with FITC as secondary antibody as described elsewhere [11]. Cells were run on a BD FACSCalibur (Becton–Dickinson, BD Bioscience, San Jose, CA) and data was analyzed using the CellQuest Pro software.

#### Statistical analysis

All data were analyzed using the GraphPad Prism program (GrapPad Software Inc., San Diego, CA). Statistical analysis of significance was undertaken by One-Way ANOVA on raw data reads using SPSS (SPSS Inc., Chicago, IL). EC_50_ values were calculated using the freeware ED50plus (v1.0) software (http://sciencegateway.org/protocols/cellbio/drug/data/ed50v10.xls).

### Results

To undertake screening of compounds for anti-DENV activity, two cell lines were selected, the human embryonic kidney cell line HEK293T/17, and the human hepatocellular cell line HepG2. Both cell lines are commonly used in analysis of DENV infection, although HET293T/17 cells do not represent a normal tissue involved in DENV infection.

**Fig. 1:**
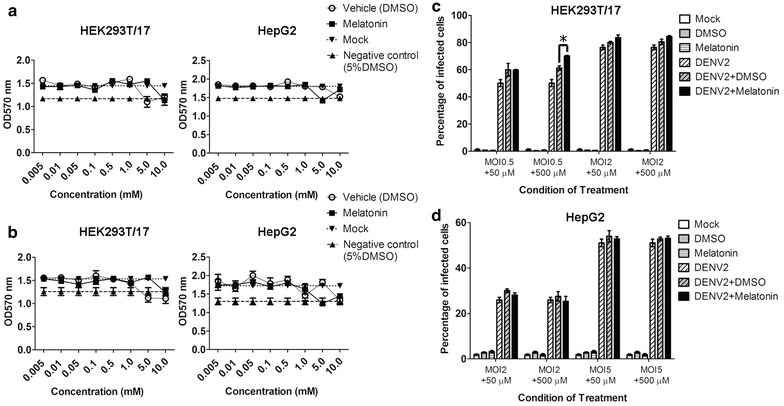
Effect of melatonin on DENV 2 infection of HEK293T/17 and HepG2 cells. The cytotoxicity of melatonin was assessed in **a** uninfected HEK293T/17 (left panel) and HepG2 (right panel) and **b** DENV 2 infected HEK293T/17 (left panel and HepG2 (right panel) cells. Infected and uninfected cells were incubated with various concentration of melatonin for 24 h and viability was assessed using MTT assay. The experiments were performed independently in triplicate in parallel with control treatments and mock. A negative of cells incubated with 5% DMSO in complete media was included. The standard deviation (SD) of mean are presented as error bars. **c** HEK293T/17 cells were infected with DENV 2 at MOI 0.5 and 2, and then treated with or without 50 and 500 µM of melatonin or with vehicle only. **d** HepG2 cells were infected with DENV 2 at MOI 2 and 5 and then treated with or without 50 and 500 µM of melatonin or with vehicle only. At 24 h post infection cells were analyzed by flow cytometry to determine the percentage infection. All experiments were undertaken independently in triplicate. Error bar showed mean ± SD (*p value ≤ 0.05)

**Fig. 2:**
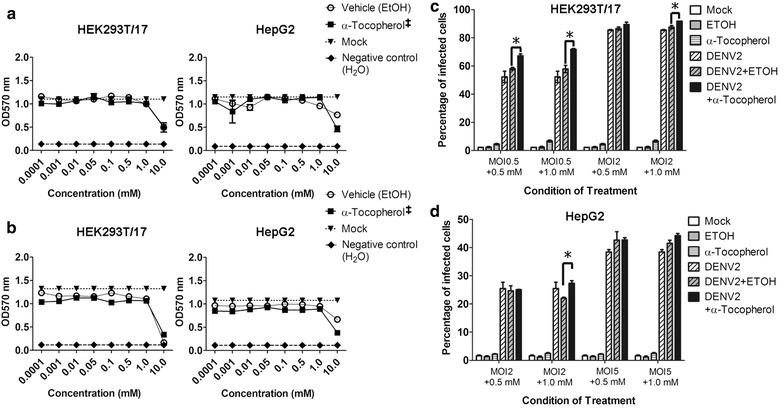
Effect of α-tocopherol on DENV2 infection of HEK293T/17 and HepG2 cells. The cytotoxicity of α-tocopherol was assessed in **a** uninfected HEK293T/17 (left panel) and HepG2 (right panel) and **b** DENV 2 infected HEK293T/17 (left panel and HepG2 (right panel) cells. Infected and uninfected cells were incubated with various concentration of α-tocopherol for 24 h and viability was assessed using MTT assay (^‡^absorbance value of α-tocopherol was removed from all samples to calculate MTT value). The experiments were performed independently in triplicate in parallel with control treatments and mock. A negative of cells plus H_2_O only was included. The standard deviation (SD) of mean are presented as error bars. **c** HEK293T/17 cells were infected with DENV 2 at MOI 0.5 and 2, and then treated with or without 0.5 and 1 mM of α-tocopherol or with vehicle only. **d** HepG2 cells were infected with DENV 2 at MOI 2 and 5 and then treated with or without 0.5 and 1 mM of α-tocopherol or with vehicle only. At 24 h post infection cells were analyzed by flow cytometry to determine the percentage infection. All experiments were undertaken independently in triplicate. Error bar showed mean ± SD (*p value ≤ 0.05)

**Fig. 3:**
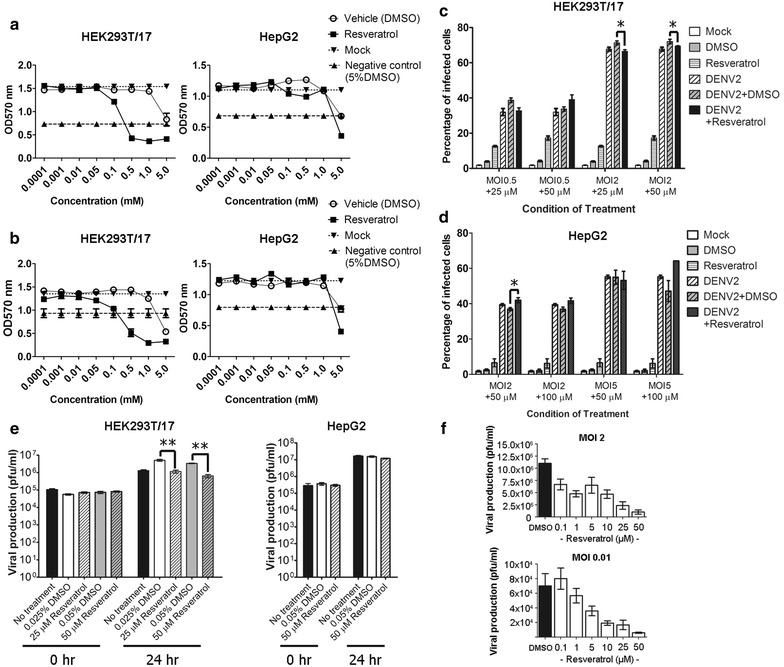
Effect of resveratrol on DENV2 infection of HEK293T/17 and HepG2 cells. The cytotoxicity of resveratrol was assessed in **a** uninfected HEK293T/17 (left panel) and HepG2 (right panel) and **b** DENV 2 infected HEK293T/17 (left panel and HepG2 (right panel) cells. Infected and uninfected cells were incubated with various concentration of resveratrol for 24 h and viability was assessed using MTT assay. The experiments were performed independently in triplicate in parallel with control treatments and mock. A negative of cells plus 5% DMSO in complete media was included. The standard deviation (SD) of mean are presented as error bars. **c** HEK293T/17 cells were infected with DENV 2 at MOI 0.5 and 2, and then treated with or without 25 or 50 μM resveratrol or with vehicle only. **d** HepG2 cells were infected with DENV 2 at MOI 2 and 5 and then treated with or without 50 or 100 μM resveratrol or with vehicle only. At 24 h post infection cells were analyzed by flow cytometry to determine the percentage infection. All experiments were undertaken independently in triplicate. Error bar showed mean ± SD (*p value ≤ 0.05). **e** For infections showing a significant difference in infection from **c** and **d**, the supernatants at 0 h (input virus; 0 h) and 24 h post infection (24 h) were assayed for virus titer by standard plaque assay. **f** HEK293T/17 cells were infected with DENV 2 at MOI 2 or 0.01 and treated with increasing concentrations of resveratrol or treated with vehicle and at 24 h post infection supernatants were assayed for virus titer by standard plaque assay. All experiments were undertaken independently in triplicate. Error bar showed mean ± SD (*p value ≤ 0.05, **p value ≤ 0.01)

Five natural products (melatonin, α-tocopherol, folic acid, acetyl-l-carnitine and resveratrol) were selected for screening for possible anti-DENV 2 activity. All compounds were initially screened for cytotoxicity in both cell lines using the MTT assay. Cytotoxic effects were determined for both uninfected cells and DENV 2 infected cells in the range of 0.0001–10 mM (0.1–10,000 μM) for acetyl-l-carnitine, melatonin and α-tocopherol, and the range of 0.0001 mM (0.1–5000 μM) for folic acid and resveratrol, with treatment being undertaken for 24 h. Results showed little difference in cytotoxicity profiles of the compounds between DENV 2 infected and uninfected cells. Thus there was no cytotoxic synergism between the compounds and DENV infection. Melatonin (Fig. 1a, b), α-tocopherol (Fig. 2a, b) and acetyl-l-carnitine (Additional file 1: Figure S1A, B) all showed evidence of cytotoxicity at compound concentrations of 10 mM. Folic acid (Additional file 2: Figure S2A, B) and/or the vehicle showed a slight positive effect in both HEK293T/17and HepG2 cells at concentration of 1 mM, but this was not seen at a higher concentration. Cytotoxicity was observed for resveratrol at concentrations above 0.05 mM in HEK293T/17 cells, and above 1 mM in HepG2 cells (Fig. 3a, b). Based on the cytotoxicity profiles, two concentrations were selected for each compound for assay of antiviral activity, which were 50 and 500 μM for acetyl-l-carnitine and melatonin, 0.5 and 1.0 mM for α-tocopherol, 0.1 and 0.5 mM for folic acid. For resveratrol which showed significant cell type variation in cytotoxicity, values of 25 and 50 μM were used for HEK293T/17 cells, and 50 and 100 μM were used for HepG2 cells. For all experiments, infections were undertaken at MOI 0.5 and 2 for HEK293T/17 cells and 2 and 5 for HepG2 cells due to different cell susceptibility to infection [12].

#### Melatonin

Melatonin (*N*-acetyl-5-methoxy tryptamine) is a natural hormone that is primarily produced by the pineal gland and regulates the circadian rhythm [13], and exogenous melatonin is often administered to adjust the sleep–wake cycle [14]. However, studies have shown that melatonin also has significant antiviral effects. Melatonin has been shown to have some effects in animal models of Venezuelan equine encephalitis virus [15, 16], Semliki Forest virus [17], West Nile virus [17], respiratory syncytial virus [18] and its use has been proposed in humans infected with Ebola virus [19]. In this study, melatonin showed a slight proviral effect upon DENV 2 infection in HEK293T/17 cells (Fig. 1c) under conditions of the lowest MOI used, and the highest concentration of melatonin, but no effect was seen in HepG2 cell (Fig. 1d).

#### α-Tocopherol

Vitamin E refers to compounds classed as both tocopherols and tocotrienols. There are four vitamin E tocopherols, termed α-, β-, γ- and δ-, and while γ-tocopherol is the most abundant form of vitamin E, α-tocopherol is the second most abundant and the most biologically active form [20]. Vitamin E has well known antioxidant properties, through its ability to donate a hydrogen group from its free hydroxyl group to the free radical, with the generation of a stable free radical form of vitamin E. However, a number of non-antioxidant activities of vitamin E have been demonstrated [21]. α-Tocopherol has been reported to have some effects in dengue patients [22] and has proposed benefits for influenza virus A infection [23], as well as possible activity against hepatitis B [24] and C [25]. In this study, α-tocopherol showed proviral effects, particularly in HEK293T/17 cells (Fig. 2c), where the percentage of infection was increased significantly under most condition combinations tested. However, only a small effect was seen in HepG2 cells under conditions of the highest concentration and lowest MOI tested (Fig. 2d).

#### Acetyl-l-carnitine

Acetyl-l-carnitine (ALCAR) is the acetylated form of l-carnitine a molecule naturally produced by the body and the acetylated form of l-carnitine is able to cross the blood brain barrier [26]. l-Carnitine functions primarily to transport activated long chain fatty acids into mitochondria for degradation by β-oxidation, and β-oxidation has been proposed to be important in DENV replication [27]. In addition, l-carnitine has been shown to have activity against hepatitis C virus [28]. In this study, ALCAR was shown to have no effect on DENV 2 infection in either cell line (Additional file 1: Figure S1C, D).

#### Folic acid

Folic acid is the synthetic form of vitamin B9, while folate is the naturally occurring form found in food. Folic acid is essential for nervous system development, and is a commonly administered supplement to pregnant mothers to prevent neural tube defects [29]. Folic acid is also important in the synthesis of RNA and DNA, and is particularly important in rapidly dividing cells [30]. Folic acid additionally plays a role in the formation of red blood cells [30]. Folic acid has not previously been explored for anti-viral activity, although the metabolically active form of folic acid, folinic acid has been described as having pro-flaviviral effects through the thymidine synthesis pathway [31]. In this study, folic acid had little effect upon DENV 2 infection in either cell line (Additional file 2: Figure S2C, D), although a slight proviral effect was seen at the highest concentration examined in HepG2 cells (Additional file 2: Figure S2D).

#### Resveratrol

Resveratrol (3,5,4′-trihydroxy-trans-stilbene) is a hydroxylated derivative of stilbene, and a phytoalexin which is produced in response to pathogen attack in some plants. It is found in nature in several foods including grapes and blueberries [32]. Considerable attention has been focused on resveratrol for potential anti-cancer [33], anti-obesity [34], anti-neurodegeneration [35] and cardio-protective activities [32]. Studies have shown that resveratrol has activity against a number of viruses including HIV [36], Middle East respiratory syndrome coronavirus (MERS-CoV) [37], influenza A virus [38], poxvirus [39] and enterovirus 71 [40].

As noted earlier, resveratrol showed significant cytotoxicity in HEK293T cells, but that HepG2 cells were somewhat less sensitive to the cytotoxic effects of the compound (Fig. 3a, b). However, infection with DENV 2 in the presence of resveratrol (at 25 and 50 μM) in HEK293T/17 cells showed a significant reduction in the level of infection (Fig. 3c), while a slight increase in infection levels was seen in HepG2 cells (Fig. 3d) with the lowest MOI and 50 μM resveratrol. We observed some background in the flow cytometry with resveratrol (Fig. 3) and this might be related to the protein binding ability of resveratrol [41]. However, subtraction of the background did not alter the result. To further investigate the effect of resveratrol in DENV 2 infection, the effect on virus production was determined by standard plaque assay. The virus titer was determined at 0 h (input virus) and at 24 h post infection. The results (Fig. 3e) showed that, consistent with the reduction in the level of cellular infection (Fig. 3c), there was a reduction in the level of virus produced with treatment with resveratrol in HEK293T/17 cells at 24 h post infection, but not in HepG2 cells (Fig. 3e). The reduction of virus output observed was the order of approximately 1log_10_. To determine the EC_50_ of resveratrol on virus production, the experiment was repeated in HEK293T/17 cells with concentrations of 0, 0.1, 1, 5, 10, 25 and 50 μM resveratrol. Results of the standard plaque assay are shown in Fig. 3f. The EC_50_ value was determined as 24.37 μM. Finally, the experiment was undertaken at a lower MOI of 0.01. Results (Fig. 3f) showed a dose dependent reduction of virus production, with an EC_50_ value of 11.37 μM. While the reduction in level of infection and virus output is significant in HEK293T/17 cells, it compares poorly to other natural compounds such as andrographolide, which reduces DENV 2 virus output by 2.5log_10_ [11].

### Conclusion

These results suggest that some commonly taken natural compounds may have beneficial effects on DENV infection, but that others may potentially add to the disease burden.

## Limitations

1. Only one of the four DENV serotypes was examined.
2. Cytotoxic effects limited the concentration test range of compounds.

## Additional files

**Additional file 1.** Effect of acetyl-l-carnitine on DENV2 infection of HEK293T/17 and HepG2 cells.

**Additional file 2.** Effect of folic acid on DENV2 infection of HEK293T/17 and HepG2 cells.

## Abbreviation

DENV: dengue virus
