# Supplementary material for: Screening of melatonin, α-tocopherol, folic acid, acetyl-l-carnitine and resveratrol for anti-dengue 2 virus activity
Source: BMC Res Notes. 2018 May 16;11:307. doi: 10.1186/s13104-018-3417-3 (PMC5956857; doi:10.1186/s13104-018-3417-3)
Supplement: Supplementary file 2 — Additional file 2. Effect of folic acid on DENV2 infection of HEK293T/17 and HepG2 cells. [file 13104_2018_3417_MOESM2_ESM.pdf]

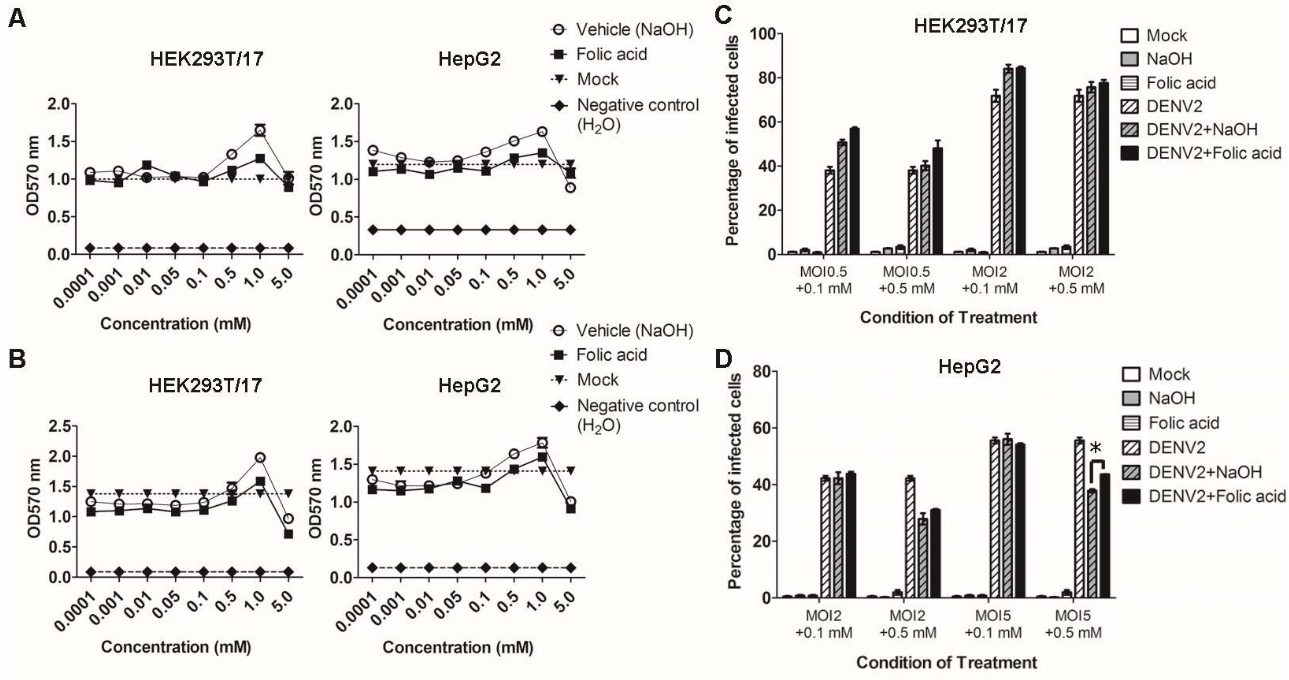

Figure S2. Effect of folic acid on DENV2 infection of HEK293T/17 and HepG2 cells.

The cytotoxicity of folic acid was assessed in (A) uninfected HEK293T/17 (left panel) and HepG2 (right panel) and (B) DENV 2 infected HEK293T/17 (left panel and HepG2 (right panel) cells. Infected and uninfected cells were incubated with various concentration of folic acid for 24 hours and viability was assessed using MTT assay. The experiments were performed independently in triplicate in parallel with control treatments and mock. A negative of cells plus H_2O only was included. The standard deviation (SD) of mean are presented as error bars. (C) HEK293T/17 cells were infected with DENV 2 at MOI 0.5 and 2, and then treated with or without 0.1 and 0.5 mM folic acid or with vehicle only. (D) HepG2 cells were infected with DENV 2 at MOI 2 and 5 and then treated with or without 0.1 and 0.5 mM folic acid or with vehicle only. At 24 hours post infection cells were analyzed by flow cytometry to determine the percentage infection. All experiments were undertaken independently in triplicate. Error bar showed mean ± SD. (*; p value ≤ 0.05).
